# Comparing prothrombin complex concentrate and fresh frozen plasma with blood viscosity characteristics in patients with trauma-induced coagulopathy

**DOI:** 10.1186/cc14430

**Published:** 2015-03-16

**Authors:** O Tarabrin, I Tyutrin, S Shcherbakov, D Gavrychenko, G Mazurenko, V Ivanova, P Tarabrin

**Affiliations:** 1 Odessa National Medical University, Odessa, Ukraine

## Introduction

To compare the effectiveness of prothrombin complex concentrate and fresh frozen plasma (FFP) in patients with multiple injuries, complicated with coagulopathy bleeding.

## Methods

The study involved 51 patients who entered Odessa Regional Hospital with traumatic injuries (concomitant skeletal trauma) complicated with hypocoagulation. Patients were divided into two groups: in the first group (26 patients), as a treatment for coagulopathy, was administered PCC in a dose of 1 ml/kg (25 IU/kg); in the second group (25 patients) was administered FFP in a dose of 15 ml/kg. Evaluation of the functional state of the hemostasis system was carried out using low-frequency thromboelastography (LFTEG) on admission to hospital and 24 hours after the patient's admission to the ICU.

## Results

According to LFTEG, polytrauma patients had statistically significant abnormalities in platelet aggregation (intensity of contact coagulation (ICC)), in coagulation (intensity of coagulation drive (ICD), clot maximum density (MA)) and in fibrinolytic activity (index of retraction and clot lysis (IRCL)). ICC in patients with multiple injuries was decreased by 27.51%, ICD was decreased by 34.68%, MA was decreased by 75.16%, IRCL was 91.06% above the norm. Patients of group 1 according to LFTEG had significant changes in all parts of coagulation 24 hours after intensive care. Indicators of platelet hemostasis characterized by persistence of hypoaggregation: ICC was decreased by 24.51%, compared with the norm; parameters of coagulation and fibrinolysis had a reliable trend toward normal and decreasing the activity, the fibrinolysis index reached normal reference values. Patients in group 2 had hypoaggregation and hypocoagulation state with increased activity of fibrinolysis: ICC was reduced by 25.62%, ICD decreased by 19.76%, MA was decreased by 22.34%, IRCL was increased by 24.52%. Clinically, patients of group 1 had reduced indicators for infectious complications, reducing the term of mechanical ventilation and reducing the volume of blood transfusions.

## Conclusion

Patients with multiple injuries have violation in all parts of blood coagulation. The use of prothrombin complex concentrate can reduce the severity of pathological changes in the hemostatic system in patients with polytrauma.

